# From Pleistocene to Holocene: the prehistory of southwest Asia in evolutionary context

**DOI:** 10.1007/s40656-017-0152-3

**Published:** 2017-08-14

**Authors:** Trevor Watkins

**Affiliations:** 1 School of History, Classics and Archaeology, University of Edinburgh, Edinburgh, UK; 2 School of History, Classics and Archaeology, William Robertson Wing, Old Medical School, Teviot Place, Edinburgh, EH8 9AG UK

**Keywords:** Epipalaeolithic, Neolithic, Southwest Asia, Cultural niche construction theory, Cognitive-cultural co-evolution

## Abstract

In this paper I seek to show how cultural niche construction theory offers the potential to extend the human evolutionary story beyond the Pleistocene, through the Neolithic, towards the kind of very large-scale societies in which we live today. The study of the human past has been compartmentalised, each compartment using different analytical vocabularies, so that their accounts are written in mutually incompatible languages. In recent years social, cognitive and cultural evolutionary theories, building on a growing body of archaeological evidence, have made substantial sense of the social and cultural evolution of the genus Homo. However, specialists in this field of studies have found it difficult to extend their kind of analysis into the Holocene human world. Within southwest Asia the three or four millennia of the Neolithic period at the beginning of the Holocene represents a pivotal point, which saw the transformation of human society in the emergence of the first large-scale, permanent communities, the domestication of plants and animals, and the establishment of effective farming economies. Following the Neolithic, the pace of human social, economic and cultural evolution continued to increase. By 5000 years ago, in parts of southwest Asia and northeast Africa there were very large-scale urban societies, and the first large-scale states (kingdoms). An extension of cultural niche construction theory enables us to extend the evolutionary narrative of the Pleistocene into the Holocene, opening the way to developing a single, long-term, evolutionary account of human history.

## Introduction: three strands twisted in one thread

As an archaeologist specializing in the Neolithic period in southwest Asia (the first three or four millennia of the Holocene, approximately 12,000–8000 years ago), I have been frustrated that most accounts of human evolution end with the emergence of Homo sapiens, or with the emergence of fully modern language, ‘art’, or ’modern’ human minds around 40,000–30,000 years ago. Palaeolithic specialists and evolutionary scientists seem to find it very difficult to carry their accounts beyond the Pleistocene, and into the Holocene. The scale of things—large, dense settlements, cities, kingdoms, empires—and the sudden and massive acceleration in the pace of cultural and social evolution perhaps seems scarily different from the evolutionary pace of the Pleistocene; cultural history was perhaps assumed to have become the appropriate narrative, and biological evolution to have become an irrelevant theme. Gene-culture co-evolution and cultural niche construction theory, however, are showing the way forward.

But I have also been frustrated that most of my colleagues working on ‘neolithisation’—the processes of transformation within the Neolithic period—tend to focus exclusively on that Neolithic period; indeed, most of them are concerned only with the early part of the Neolithic period. Despite its pivotal role in human history, the Neolithic has become effectively disconnected from the grand narrative in which it is said to be pivotal. I am now experimenting with cultural niche construction theory as a conceptual framework that allows us to develop an evolutionary narrative that crosses that Pleistocene-Holocene divide. In principle, once we have an evolutionary narrative that makes sense of the Neolithic transformation, we may expect to be able to extend that narrative to link the last two or three millennia of prehistory into the beginnings of the historical states, kingdoms, and empires of ancient history; but this is beyond the scope of this paper.

Most people think of the Neolithic in terms derived from Gordon Childe’s theory of a Neolithic revolution (Childe 1936). He defined it as a period of a kind with the industrial revolution, bringing about a social, economic and technological transformation. It was the time when the age-old world of mobile foraging bands was supplanted by a new world of village-farmers. The domestication of plants and animals and the spread of farming have been major focus of much innovatory and interdisciplinary research, and it is in those economic and biological terms that archaeologists have commonly defined the period since Childe’s time. However, I see it differently. I see the process leading to and through the Neolithic as three strands that are twined together to make one thread; and that thread begins a long time before the Neolithic period. Let me introduce these three strands and show how each tells part of a story that we can follow from at least the Middle Palaeolithic-Upper Palaeolithic transition around 40,000 years ago. Then, I will seek to set this major transformation process in an evolutionary framework.

### Intensification of subsistence strategies

One strand indeed concerns changes in subsistence strategies, culminating in the establishment of effective mixed farming practices. From 40,000 years ago onwards increasing numbers of heavy ground stone implements that were used for grinding and pounding dry grasses, cereals and legumes have been found on archaeological sites in the Levant (the only part of southwest Asia that has been sufficiently explored to give us coherent information of this kind). Recent research has recovered starch traces from the surfaces of heavy stone grinding implements at several sites in different parts of Europe dating around 30,000 years ago, shortly before the Last Glacial Maximum (Mariotti Lippi et al. 2015; Revedin et al. 2010). From the site of Ohalo II, on the southern edge of the Sea of Galilee, there is direct evidence of the seeds of as many as 120 species of grasses, cereals and legumes by 23,000 years ago (Nadel et al. 2012; Piperno et al. 2004; Weiss et al. 2004). People were beginning to make greater investments in time and heavy labour in harvesting, storing, and processing these plant foods, and taking a delayed return perspective. Around the beginning of the Neolithic—that is, around 11,500 years ago—some groups began to cultivate some of these plant species (Willcox et al. 2008; Willcox and Stordeur 2012). As cultivation intensified, the spectrum of species was narrowed down, and, over many centuries, domesticated forms of wheat and barley, peas, beans, and lentils became the established, staple crops (Colledge et al. 2004; Coward et al. 2008; Willcox et al. 2012). The beginnings of farming can be seen to have a long prehistory; and farming became effective only two millennia (or more) after cultivation began.

There is evidence that human population density in the southern Levant increased steadily from the Middle-Upper Palaeolithic transition (see below). And it seems that increased hunting reduced the populations of their preferred prey (Davis 1981, 2005). The largest species had practically disappeared by 40,000 years ago. Next, red deer, and then fallow deer became rare, and hunters concentrated more and more on the much smaller gazelle. As larger prey became more difficult to access, and as people tended to become more sedentary and less mobile, there is a discernible trend towards more intense exploitation of small mammals, such as fox and hare, and birds, reptiles, amphibians and fish (Stiner and Munro 2002; Stiner et al. 2000). People were investing greater time and effort, skill and equipment for smaller returns. The first domesticated sheep, goat, cattle and pigs begin to appear soon after 10,500 years ago, more than a 1000 years into the Neolithic, and over the following two millennia herded animals gradually replaced hunted species (Conolly et al. 2011).

### Increasing population density, increasing size of co-resident groups

The second strand is a remarkable demographic phenomenon. In their survey that sets the Neolithic throughout the Levant within a longer-term perspective, Goring-Morris and Belfer-Cohen (2011) used the number of archaeological sites per 1000 years as a proxy indicator of population numbers. The best data comes from the southern Levant, where most fieldwork has been concentrated over many decades; the increase in the number of sites, period by period, from the Upper Palaeolithic (between 40,000 and 25,000 years ago) onwards, forms a dramatic upward curve. There is much less data for the northern Levant, that is, northwest Syria and southeast Turkey, particularly for the Upper Palaeolithic and Epi-palaeolithic periods. The data for the Neolithic, however, shows a similar upward curve.

Not only does the number of sites grow through the Neolithic, but their area shows an exponential increase by a factor of 10 over less than 4000 years, as demonstrated by Ian Kuijt (2000) on the basis of the data from the better-documented southern Levant. Kuijt also noted that the density of buildings on sites quadrupled over that period, and domestic buildings increased in scale and compartmentalization. The combined effect of more and larger settlements, made up of larger, more complex buildings many of them two-storied, packed into the space to the maximum extent, implies a growth in the size of population units by a factor of 60, or even 100 times. Settlements existed over many centuries, and comprised populations of many hundreds, and in some cases more than 5000 people.

### Imagery, monuments, acts of symbolic representation

The third strand concerns the powerful material imagery and monuments of the early Neolithic. The new, permanent settlements were not just clusters of huts. The earliest Neolithic settlements, such as Qermez Dere in northern Iraq, were structured; the living areas were segregated from the area where all the waste materials and rubbish were deposited (Watkins 1990). A number of these earliest settlements have been found to possess elaborate and monumental public buildings. Even the small buildings were treated with extraordinary care throughout their use-lives; and at the end of that use-life buildings might be ceremonially burned and buried.

Some of the most striking, and genuinely monumental, examples of public buildings have been found in the course of salvage excavations in settlements in the Euphrates valley. The most extensively investigated of those settlements was Jerf el Ahmar (Stordeur 2015), which was occupied for almost a millennium around 11,000 years ago. Over that long time, as old buildings were repeatedly replaced, the village footprint drifted, and at least four, conspicuously larger, circular, semi-subterranean public buildings succeeded one another. The excavator, Danielle Stordeur, has called them communal buildings (‘bâtiments communautaires’) (Stordeur 2015; Stordeur et al. 2000). Very similar monumental, circular, subterranean buildings have been found at other contemporary settlements in the region; for example, a series of several was found at Tell ‘Abr 3, also in the Euphrates valley (Yartah 2005, 2016). Another, rather different, large, semi-subterranean, circular building lies at the centre of the contemporary settlement of WF16, in Wadi Feynan, southern Jordan (Finlayson et al. 2011; Mithen et al. 2011). And a contemporary settlement on Cyprus, Ayios Tychonas-Klimonas, has been found to have another example (Vigne et al. 2011, 2012).

When considering the best preserved of the Jerf el Ahmar examples, the scale of the communal effort involved in creating one of these subterranean structures is striking (especially by any archaeologist with digging experience). The cavity that was dug is 7 m in diameter and more than 2 m deep, requiring almost 200 cubic metres of soil to be removed before construction began. There is evidence that the building within the cavity was reconstructed more than once. The interior of its last form comprised a set of doorless cells, and an open area with packed earth floors that formed a series of low platforms. The remains of stores of grain and lentils were found in some of the cells (Stordeur and Willcox 2009), but the function of the open area is unknown. At the end of its life, the decapitated body of a young woman was dropped on the floor (the only access to the building being by means of an aperture in the roof), the roof was set on fire, and finally the void was completely filled in—just like the houses at Qermez Dere.

The most strikingly monumental structures are the enormous circular enclosures at Göbekli Tepe in southeast Turkey (Dietrich et al. 2012; Notroff et al. 2014; Schmidt 2006, 2010, 2011, 2012). They share a number of features with the communal buildings at the settlements in the Euphrates valley, including the repeated attention to modifying or re-working, and their final deliberate burial. But although it looks like a settlement mound, Göbekli Tepe is not another settlement site. It is situated on a bare mountain ridge of limestone overlooking the great Mesopotamian plain. We do not yet know how the 15-17 m thick mound of cultural debris was formed, but it is clear that it did not accumulate through the same everyday processes of creating and replacing buildings and workspaces of everyday life, and disposing of domestic residues that are found in the settlement sites. What we know of so far is a series of massive, stone-built, circular structures that were formed within massive excavated cavities. They are more than 20 m in diameter, and are found buried more than 5 m deep. In the centre of each enclosure stood a pair of tall limestone monoliths; and around the perimeter wall of the enclosure 10 or 12 more monoliths were set radially in a stone ‘bench’ that echoes the bench that we encountered in the later of the communal buildings at Jerf el Ahmar. All of the monoliths are shaped like a capital letter T. It is clear that the T-shaped monoliths are highly schematic anthropomorphs, because some of them are portrayed with human arms and hands, and two of them, the pair of central monoliths in Enclosure D, have collars round their necks, from which are suspended pendants, and decorated belts around their ‘waists’, from which hang the skins of foxes.

The imagery and symbolism at Göbekli Tepe is extraordinarily complex, and it is recursively structured, as our modern languages use recursion, setting phrases in clauses, and clauses within sentences, and so on. There are now more than a hundred such monoliths, between 3 and 5.5 m tall, many of them carrying images of wild, male animals (typically bulls, foxes, wild boar), birds, snakes, scorpions and spiders, carved in raised relief. There are, then, symbols borne by monoliths; the monoliths are set within an enclosure in a formal relationship with one another, and with the circular framework of the enclosure. At the next level, the enclosures relate to one another within the site in ways that are yet to be investigated; and, at the highest level, the enclosures end up buried and embedded within the encompassing mound, which is more than three hundred metres in diameter.

Perhaps the most unexpected artefacts to come from Göbekli Tepe, Jerf el Ahmar, and other settlement sites in the region are small stone plaquettes with motifs that are incised on their flat surfaces. There is a more common class of stone object, called by archaeologists shaft straighteners (by the French, pierres à rainure), a number of which have incised designs on the curved surface (the other surface being flat, with a groove across it, in which the arrow-shaft was abraded). However, the new group of small stone plaquettes, flattened on both surfaces, bear motifs that have all the appearance of being groups of signs. A number of the signs are simplified, linear versions of creatures that appear carved in relief on the Göbekli Tepe monoliths. The Egyptologist and semiologist Ludwig Morenz believes that these are examples of a non-textual, or semasiographic, writing system that was shared by communities across the region (Morenz 2009, 2014; Morenz and Schmidt 2009). The fact that these small, sign-bearing plaquettes have now been found at a number of the earliest Neolithic sites in north Syria and southeast Turkey, including at Göbekli Tepe, and that some of the same signs are found at several sites, deepens the kind of sharing and exchange in which these communities engaged. We know that they exchanged materials (for example, obsidian), and things (for example, carved and decorated chlorite vases, exotic marine shells and malachite beads), and shared ideas (such as the preferred shapes of arrowheads, or the design of the large communal buildings) (Watkins 2008, in press). Now we begin to see that they also shared a repertoire of ideograms (that is, semasiographic as opposed to logographic signs). The quality, intensity and extent of early Neolithic networking are of a different order from that of classic Upper Palaeolithic times.

Although these signs and the symbolic architectural and sculptural representations are new and completely unexpected discoveries, they must somehow relate to a long-term history of the making of signs, signification and material representations. At present, we lack knowledge of the immediately preceding Epi-palaeolithic period in the region where this dramatic Neolithic symbolic repertoire has been found; the relatively well-known Epi-palaeolithic of the southern Levant is not very helpful in this regard, just as the earliest Neolithic lacks the dramatic imagery and, with the exception of the massive round tower, wall and rock-cut ditch at Jericho, the monumental symbolic architecture.

## A transformation of the cultural niche

How should we understand the extraordinary explosion of novel features of the Neolithic of southwest Asia? Since Flannery’s (1969) formulation of a broad spectrum revolution archaeologists have gradually recognized that the preceding Epi-palaeolithic period, between 23,000 and 12,000 years ago, saw the emergence and development of many of the characteristics that we think of as Neolithic. But the two explanations that have competed to be the standard account have both focused on the changes in subsistence strategy that brought about domestication of plants and animals and the origins of farming practices. Binford (1968) hypothesised that human population growth required people to intensify the productivity of the environment; since the discovery of the Younger Dryas phase at the end of the Pleistocene, the alternative hypothesis has been argued on the basis of pressure on wild food resources consequent on the suddenly cooler climate (e.g. Bar-Yosef and Meadow 1995; Moore and Hillman 1992). Neither of these hypotheses accounts for the emergence of large-scale, permanent settlement or the elaboration of symbolic material culture at all scales of which we have learned from recent discoveries, and the implications of huge investments of time, labour and creative endeavour that underpin these impressive Neolithic phenomena.

I believe that cultural niche construction theory can serve as the conceptual framework that enables us to develop an evolutionary narrative reaching from the arrival of Homo sapiens in southwest Asia and making sense of the process of transformation that is associated with the Neolithic. Niche construction theory (Laland et al. 2000; Odling-Smee et al. 2003) is exciting evolutionary scientists, because it recognizes the role of organisms in contributing to the making of their selective environment, and therefore the selective environment of successive generations. Whereas conventional evolutionary theory (now referred to as Standard Evolutionary Theory—SET) has seen the environment as exerting selective pressure on a population of organisms, niche construction theory models the evolutionary process as a two-way process. It is a central feature of what has been termed the extended evolutionary synthesis (Laland et al. 2015). Given that there was already considerable scientific interest in gene-culture co-evolution, it is not surprising that some of the best examples of such co-evolution are readily situated within a cultural niche construction version of the niche construction theory (Laland et al. 2010; Laland and O’Brien 2011).

Thus, it has been recognised that modern humans and their hominin ancestors have been very powerful niche constructors because of their capacity for modifying their environment by cultural means (e.g. Smith 2011; Zeder 2009, 2012). Neolithic (farmed) landscapes had very significant knock-on effects on natural plant and animal populations, as well as on processes, such as erosion, deforestation, slopewash, and alluviation; as population density increased, and as farming populations expanded and colonised new land, their impact on the natural environment increased in effect and extent. The domestication of plants and animals brought about the emergence of new species; in the case of cereals, for example, domestication produced wheat and barley that were easier to harvest, were more productive, and easier to thresh; on the other hand, the domesticated forms were practically incapable of reproducing themselves, and needed humans to broadcast seed saved from one crop to sow the fields for the next crop. Living together in permanent settlements in close proximity with their domesticated animals meant that diseases had the opportunity to jump across species and affect humans, who had to evolve some degree of immunity in response. Gene-culture co-evolution consequent on the domestication of plants and animals has had very significant effects on the human genome, just as it has transformed cultures in many ways (e.g. O’Brien and Laland 2012). A conspicuous example is the emergence of lactase persistence in populations that have been reliant on herding and dairy products (Gerbault et al. 2011; Itan et al. 2009). In fact, gene-culture co-evolution within the cultural niche over the last 10000 years has produced extensive and rapid evolution in the human genome (e.g. Cochran and Harpending 2007).

The evolutionary theorist Kim Sterelny (2003, 2011) has been particularly concerned with the evolution of human social learning and what he has called the apprentice model. He has been an early adopter of cultural niche construction theory as a mode of unravelling the interconnections and feedback loops between human culture and the cognitive environment. Niche construction theory talks about ‘ecological engineering’ and the ‘ecological inheritance’ that is passed down to succeeding generations. Sterelny has talked of ‘epistemic engineering’ within the cultural niche, and the formation of a cultural inheritance. Critically important in human cultural niche construction is what Sterelny in his apprentice learning model has called ‘cumulative downstream epistemic engineering’, that is, the capacity so noticeable in modern Homo sapiens not only to transfer a large and complex body of cultural knowledge from generation to generation, but also to build on that inherited cultural knowledge through refinement and innovation. He has described how epistemic engineering affects human cognitive competence, and how humans organize their physical environment in ways that enhance information processing capacities.

Sterelny and Watkins collaborated to confront and, at least to some extent, resolve the Holocene challenge (Sterelny and Watkins 2015). For Sterelny, it was an exercise to carry forward his cultural niche construction programme beyond the Pleistocene, and for Watkins it was the means to set the Neolithic into its Pleistocene evolutionary context. Key to understanding the complex process, I believe, is setting the rapid population growth of the Epi-palaeolithic and Neolithic periods, and the emergence of relatively large, permanently co-resident communities into the context of what Robin Dunbar and his colleagues have called the evolution of the social brain (Dunbar 1993, 1998, 2014; Dunbar et al. 2010, 2014; Gamble et al. 2014). The social brain hypothesis observes that brain size in primates correlates with social group size; it therefore proposes that the expansion of hominin brains, and especially the expansion of the pre-frontal cortex, over evolutionary time has supported expansion in social group size, arguing that the cognitive demands of human sociality place a constraint on the number of individuals that can be maintained in a coherent group (Dunbar 2009).

## Cultural accumulation, transmission and innovation

There are good evolutionary reasons for humans to live in large social and cultural groups. Early in hominin evolution there were benefits such as security and defence, and in big-game hunting. In the more recent chapters of human evolution, particularly since the emergence of *Homo sapiens*, there are significant cultural benefits of scale and cooperation in large-scale social units (Richerson et al. 2009; Shennan 2001; Sterelny 2011, 2015); correspondingly, it is difficult, or even impossible, for social groups that reduce in size for whatever reason to sustain a large and complex body of cultural knowledge, and transmit it securely across generations (Henrich 2004). But the rate at which the size of population units increased through Epi-palaeolithic and early Neolithic was much faster than the biological brain could evolve; these groups would no longer be able to live together by means of the psychological capacities of the evolved Homo sapiens brain. Rather, there was rapid evolution of the capacity for cultural niche construction and the symbolic cultural means to construct large and robust communities.

As size of population unit increased the potential benefits in terms of having a multiplicity of experts as curators, exemplars and teachers of cultural knowledge increased apace. But the demands of cooperation grew, and the risks of free-riders and cheats likewise grew. The cultural benefits of scale were balanced by the problems of living together in large numbers among people upon whom each depended, but who were mostly little known, or unknown, to the individual. New kinds of shared norms and institutions of behaviour had to be evolved, and new ways of making community beyond the scale of natural human psychology (Watkins 2012, 2014). It is to answer these problems that we see the evolution of massive monument building and sophisticated systems of symbolic representation in material form—sculptures, signs, among others. The collective achievement of creating and maintaining the monuments allowed very large communities to share a common, governing ideology; and, at the same time, they functioned as the means to demonstrate forms of ‘commitment mechanism’, and ‘costly signalling’ (Gintis et al. 2001; Soler 2012; Sterelny and Watkins 2015), whereby the members of the community could assure themselves of the shared commitment of everyone else.

At the same time, the symbolic architecture and the imagery of the sculptures embodied worlds of meaning (Watkins 2004), constituting what Merlin Donald (1991) has called systems of ‘external symbolic storage’. The classic and familiar form of external symbolic storage is in the form of the written (or printed) word; our libraries—and now the internet and the world-wide web—are evidence of the phenomenal capacity of such systems of external symbolic storage not only to store, but also to share, practically limitless amounts of information and ideas. But elsewhere Donald has discussed how modern humans have learned to use art and architecture in a similar way to embody, store and transmit information and ideas (Donald 2006, 2009). Throughout his work, Donald has repeatedly argued that these systems of external symbolic storage that humans have created feed back into human cognition, not simply at the superficial level of giving us access to the information that they store, but also in the way that, for example, learning to read actually re-structures the operational capacity of the human brain. Inspired by the work of Merlin Donald, my hypothesis is that the development of advanced modes of external symbolic storage in material form constituted a qualitative change in the human capacity for cultural niche construction; and it was this transformation of the cognitive-cultural niche that in turn allowed people to construct in symbolic modes the new kinds of large, permanent communities.

## Making monuments, framing concepts, constructing worlds of meaning

Between 22,000 years ago, at the height of the Last Glacial Maximum, when archaeologists date the beginning of the Epi-palaeolithic period in southwest Asia, and 9000 years ago, people changed from living in small, mobile foraging groups of fluid membership to living together in large, permanently co-resident communities of many hundreds or some thousands of people, dependent on the crops that they farmed and the flocks of animals that they herded. The Palaeolithic foraging bands were the constituent elements in a fission-fusion mode of living (Grove et al. 2012; Grove and Dunbar 2015), whereby the larger social group would come together occasionally (when, on ethnographic analogy, they would enjoy ceremonies, feasting, and the opportunity for social exchanges and the finding of marriage partners). In the Neolithic, the scale of the social networks had been transformed by a factor of ten or a hundred. As many people lived permanently together in a settlement as had operated in the Palaeolithic as a fission-fusion social group distributed as small, mobile foraging bands across an extensive territory. And the permanent communities were locked into even more extensive networks of sharing and exchange (Watkins 2008, in press). The challenge was to devise ways that enabled many thousands, or tens of thousands of people who did not know each other, and for the most part never even saw one another, to be confident that they shared an idea of their world and their place in it, as they also shared in commitment and collaboration in the making, maintenance and continual re-making of the monuments at a central place like Göbekli Tepe, or the communal buildings in their settlements.

The argument in this paper is that, as explained in the previous section, the larger scale of these networked super-communities supported more efficient, more resilient cumulative cultural learning, transmission and the take-up of cultural innovations; but the building and sustaining of such large-scale super-communities required a quantitative and qualitative upscaling of the modes of symbolic representation and communication that were required for ‘the symbolic construction of community’ (Cohen 1985).

We can say that, over the final Pleistocene and the beginning of the Holocene, in parts of southwest Asia the cultural niche became the cognitive-cultural niche. The kind of cultural niche construction that Sterelny (2011) and Henrich (2015) talk about enabled the young generation to learn the necessary knowledge and skills for everyday life; they would learn, for example, how to build and maintain a house. But the cognitive-cultural niche enabled people to share, and taught the young to understand, concepts such as ‘household’ and ‘home’ and ‘neighbourhood’ as institutions, how to behave within a household, and how to behave with neighbours (Watkins 2016). Within the cultural niche experienced experts could transmit knowledge about how to hunt, or how to harvest and store; but, by the beginning of the Neolithic, the cognitive-cultural niche had been evolved to give concrete form to the idea of who they were as a community, and the communal food storage buildings embodied the community’s commitment to cooperation and trust. It is probably easier for most of us to understand how the cognitive niche works if we think of it in terms of language, rather than in terms of the equally powerful but less easily recognized non-linguistic material culture.

The high-profile psychologist and theoretical linguist Steven Pinker is best known for his research on language (Pinker 1994) and cognition (Pinker 1998, 2013). He has written specifically about language and the ‘cognitive niche’ (Pinker 2010), discussing how language and cognition may co-evolve; we are able to articulate and communicate an original thought that has come into mind, but our articulation of the thought is framed in terms of a particular language. Merlin Donald (1991, 2001) explored the way that language operates (what he calls mythic culture), before moving on to what he describes as theoretical culture, in which modern human communities have devised and incorporated systems of ‘external symbolic storage’. But Donald is quite clear that infusing symbolic meaning in material form began much earlier than the first writing systems. He wrote of Upper Palaeolithic art as illustrating the beginnings of external symbolic storage. Given what we now know of the use of red ochre, the making and wearing of beads and pendants, and the ceremonial burial of a deceased member of the group, from Middle Stone Age Africa, late Middle Palaeolithic contexts in the southern Levant, and arguably from late Neanderthal contexts in Europe, we can say that making things or representations that signify something dates back two or three times further than the beginning of the Upper Palaeolithic period. However, I would distinguish between making or doing things that signify something from language-like systems of external symbolic storage; and, in the complex recursions of sculptured stones and enclosures at Göbekli Tepe, and more particularly in the vocabulary of signs carved on small stone plaquettes, I believe that we can justly speak of language-like systems of external symbolic storage.

## Summary conclusion

I have tried to show how it is possible to embed the Neolithic in an evolutionary narrative that extends the story of human cultural evolution out of the Pleistocene and into the Holocene world. I have also sketched how cultural niche construction theory can be extended in terms of a cognitive-cultural niche to accommodate the notion that modern humans have evolved a mode of cultural niche construction that effectively frames our human cognition and enables us to live in extraordinarily large numbers in super-communities where people do not know one another, but can share the extraordinary abstractions—like being a nation—that underpin our lives today.
